# Comprehensive Knowledge of HIV and AIDS among Ghanaian Adults from 1998 to 2014: A Multilevel Logistic Regression Model Approach

**DOI:** 10.1155/2020/7313497

**Published:** 2020-09-21

**Authors:** Chris Guure, Stephen Owusu, Samuel Dery, Frances Baaba da-Costa Vroom, Seth Afagbedzi

**Affiliations:** Department of Biostatistics, School of Public Health, University of Ghana, Legon-Accra, Ghana

## Abstract

**Background:**

In order for stakeholders of HIV and AIDS to effectively plan HIV prevention programs, it is expedient to assess the level of individuals' knowledge on the most common preventive methods and misconceptions of the HIV virus. This study examines the trends and determinants of comprehensive knowledge (CK) of HIV and AIDS among Ghanaians from 1998–2014.

**Method:**

The data used for this study were drawn from the Ghana Demographic Health Surveys (GDHS), 1998–2014. A separate analysis was performed on each survey-year data and GDHS pooled dataset. Additionally, both the male and female datasets were combined. The samples used for the study were 6,389, 10706, 9484, and 13784 representing 1998, 2003, 2008, and 2014, respectively. The pooled dataset consisted of 40363 responses. The Pearson chi-square test and multilevel binary logistic regression analysis were carried out to assess the association between the study variables and CK of HIV and AIDS.

**Results:**

CK of HIV and AIDS was found to be lower in women than men (29.24% vs. 37.7%) using the pooled dataset. The Greater Accra region recorded the highest percentage of CK of HIV and AIDS (44.18%), whereas the Northern region recorded the lowest (17.87%) among the 10 administrative regions in Ghana. Comprehensive knowledge of HIV and AIDS was also found to be less likely with an OR of 0.72 (95% CI; 0.65, 0.79, *p* < 0.001) among persons living in rural areas even after controlling for other study variables. There is also a decrease of CK of HIV and AIDS from 37.35% in 2008 to 32.5% in 2014. The lowest percentage (10.75%) of CK of HIV and AIDS among the four survey years was recorded in 1998.

**Conclusion:**

There are generally low levels of comprehensive knowledge among the Ghanaian adult population more especially among women. Those residing in rural areas have lower prevalence of CK of HIV and AIDS. To address some of these challenges, there is the need to intensify educational interventions more especially among women and people leaving in rural areas to reverse some of the knowledge gaps and correct the local misconceptions of HIV and AIDS.

## 1. Background

It is estimated that 76.1 million people have become infected with HIV, with about 36.7 million people living with HIV across the globe as of 2016 since the epidemic began [1]. This makes HIV and AIDS a public health concern that needs to be tackled with all seriousness. The epidemic is not only responsible for the loss of millions of lives (35 million since the start of the epidemic) but also ensures a decline in social and economic development in the affected communities [2]. The disease has been estimated to reduce economic growth rates by 2–4% a year across Africa [3]. It is having a profound burden on the insufficient resources available to countries, especially in the developing and underdeveloped countries. The impact of HIV and AIDS in the world is devastating, especially in sub-Saharan Africa, where about 70% of the world's HIV and AIDS patients live [4].

Life expectancy in the sub-Saharan African region has been on the decline since the disease found its way into the region. It has been found that high mortality, orphanhood, dependency, and lower productivity have led several low-income countries into further poverty [5]. The epidemic is also responsible for the stigmatization and discrimination of the affected individual [5]. Lastly, the HIV and AIDS epidemic has considerably increased the demand for healthcare, thereby creating a burden on the already weak health system, particularly in sub-Saharan Africa [5].

Though Ghana's incidence and prevalence rates are lower compared to other countries in the sub-Saharan region, through the successful rollout of the National Strategic Frameworks (NSF I, NSF II) and National Strategic Plan (NSP), the detrimental effect of HIV and AIDS in Ghana's economy cannot be overemphasized [6]. Efforts in addressing this epidemic need to be sustained and maintained to lower both incidence and prevalence rates. Knowledge of HIV and AIDS is relevant to ensuring safer behaviour that will help to reduce the risk of acquiring the infection. Therefore, global stakeholders are now adopting behavioral and attitudinal changes as a major tool to address this epidemic [7].

Estimate of HIV and AIDS for Ghana from 1990 to 2016 depicts a decline in the number of new infections [8]. The prevalence rate in the country was estimated to be 1.6% (adults between 15 and 49 years) [8]. Nevertheless, Ghana has been witnessing inconsistencies in prevalence rates across various regions. The regional prevalence for 2016 ranged from 2.7% in both Volta and Brong Ahafo (the highest) regions to as low as 0.7% in the Northern region [9]. Meanwhile, in 2015, the Northern region recorded a prevalence of 1.2% [10]. In the same year, the Greater Accra region had the highest prevalence of 3.2%, overtaking the Eastern region which recorded the highest in 2014 [10]. In Ghana, the data available gives an indication that general knowledge and awareness of HIV and AIDS is high. According to the Ghana Statistical Service (2015), about 98% of women and 99% of men admitted that they have heard of HIV and AIDS [6]. However, most Ghanaians seem to have inadequate CK of HIV and AIDS. It is reported that only 18% of women and 30% of men in Ghana have CK of HIV and AIDS [6]. It is in this light that this research is conducted to ascertain the factors that may influence CK of HIV and AIDS. This manuscript will also help us to have a fair picture of the trend in CK of HIV and AIDS from 1998 to 2014.

This study, therefore, seeks to answer three general questions: First, what is the trend in the comprehensive knowledge of HIV and AIDS among Ghanaians from 1998 to 2014? Second, what are the distributions of CK of HIV and AIDS among Ghanaians? Finally, what factors determine CK of HIV and AIDS among adults in Ghana?

## 2. Methodology

### 2.1. Data Source and Sample Design

This study used data from the Ghana Demographic and Health Survey from 1998–2014 for analysis. The Ghana Demographic and Health Survey (GDHS) is a nationally representative household survey that collects a very wide range of population, health, and other important indicators covering all the ten regions of Ghana. Participants in the survey were asked retrospective questions spanning 5 years prior to the survey.

All the GDHS surveys used a two-stage sampling procedure to be able to estimate indicators at the national level, rural, and urban, as well as each of the 10 administrative regions in the country. The first stage involves the selection of clusters which are made up of enumeration areas (EAs) obtained from the 2010 PHC. There were 427, 412, 412, and 400 clusters selected for 2014, 2008, 2003 and 1998 survey years, respectively. Systematic sampling was used in the second stage to conduct household interviews. A household listing was undertaken in all the selected EAs, and those included in the survey were randomly selected from the list. Within each cluster, about 30 households were selected to constitute a total of 12,831 households in 2014. Furthermore, 12,323, 6,600, and 6,375 households were selected from 2008, 2003, and 1998 survey years, respectively.

### 2.2. Dependent Variable

Comprehensive knowledge of HIV and AIDS is defined as being able to correctly answer five questions by identifying (1) two major preventive methods of HIV (using a condom and having one faithful uninfected sexual partner), (2) knowing that a healthy-looking person can be diagnosed of HIV, and (3) being able to reject two local most common misconceptions about the disease (sharing food with an individual who has AIDS and can get infected through mosquito bites) [1]. Comprehensive HIV and AIDS knowledge was coded as a binary outcome. If an individual correctly answers all five questions, the individual is considered as having CK of HIV and AIDS and, hence, a code of “1” was given. Otherwise, a code of “0” was given to an individual who could not answer at least one of the questions correctly. The measurement and computation of CK was the same across all survey years.

### 2.3. Independent Variables

DHS interviews men and women with different questionnaires. Majority of the questions are common to both men and women except for topics peculiar to women such as reproductive health, maternal and child health, and maternal mortality. Nonetheless, this study concentrates on only variables that are common to both males and females. The ages of the respondents interviewed in the GDHS surveys were numerical; however, this study grouped them into nine categories from 15 to 49 years. In addition, the residences of the respondents were categorized into rural and urban areas.

The responses of the religious affiliations were recorded. This study combined “no religion” and “other religion.” Furthermore, “Pentecostal/Charismatic” and “other Christians” were combined. Other categories included “Catholic,” “Anglican,” “Methodist,” “Presbyterian,” “Traditionalist,” and Muslims. Respondents were asked to indicate the ethnic group they belong to out of a list of the major ethnic groups in the country. The categories were recorded so that all the survey years would have analogous categories.

GDHS assessed literacy by asking respondents to read a simple sentence in their local language or in the English language. Scores were given for how each individual performed. Those who had ever been or were in secondary or higher education were exempted from the test because they were assumed to be literate. Individuals who were visually impaired were also not considered. The scores were, then, categorized into those who could read easily, those who could read with difficulty, and those who could not read at all.

Other independent variables include marital status of the respondent, highest educational attainment of respondents, media use, and the type of contraceptives used by respondents: this variable was recorded into whether they are recently using contraceptives or not.

### 2.4. Statistical Analysis

A separate analysis of the GDHS data was performed for each survey year using Stata version 15 incorporating weights through the survey analysis functions. Percentages and odds ratios stated were weighted with a 5% level of significance. Cross tabulation was used to examine associations between the outcome variable and the independent variables for each survey year (1998–2014), as well as for the pooled dataset. The Pearson chi-square test and multilevel logistic regression were used to examine the statistical significance of associations between comprehensive HIV and AIDS knowledge and potential predictors. In the Multilevel logistic regression, the binary outcome variable was coded as “not having comprehensive knowledge,” the reference category, and “having comprehensive knowledge,” and multilevel logistic regression models were also performed taking into consideration the structure of the data collection process to obtain accurate adjusted odds ratios with their corresponding confidence intervals for each survey year and pooled survey years. The research was purely on complete case analysis, and so all missing observations were removed.

### 2.5. Multilevel Logistic Regression Model

Multilevel models, also referred to as hierarchical, nested, or mixed effects models, are statistical models where parameters vary at more than one level [11]. GDHS uses a data structure where individuals are nested within households and households nested within clusters. Thus, the data have a hierarchical structure; hence, a standard logistic regression model may not be sufficient. This is because standard logistic regression is usually unable to adjust for the variation due to clustering and, hence, fails to appropriately estimate associations among responses within clusters or households. Subsequently, incorrect standard errors will be estimated. Another reason why one cannot use standard logistic regression analysis is that the independence of residuals will be violated: individuals in the same household or cluster are more likely to behave in similar characteristics, which may result in correlated responses in the same cluster. To model hierarchical data with a binary response, generalized linear mixed effects (multilevel) models are mostly used. This model comprises of a fixed effect which is analogous to the standard regression model and random effects components which explains the cluster-specific evolution [12].

For this study, level one units were individuals, both males and females, and level two units were the households to which these individuals belong to. Level three was the clusters within which households were nested.

The model with the logit link function is defined as

(1) $$\begin{array}{c} \mathrm{Logit}\;\left(P_{ij}\right)=x_{ij}^{\prime }\beta +\;\mu_{i}, \end{array}$$

where *P*_*ij*_ denotes the probability that an individual has comprehensive HIV and AIDS knowledge. *x*_*ij*_′ is a vector of observed covariates, and *β* is the vector of regression parameters and the random effects at the cluster level. *μ*_*i*_ is assumed to follow the normal distribution with mean 0 and a constant variance [13].

### 2.6. Intraclass Correlation (ICC)

The ICC computes the degree of homogeneousness of the outcome variable within clusters [12]. It represents the proportion of the between-cluster variation in the total variation. The ICC ranges from 0 to 1. An ICC of 0 indicates perfect independence of residuals: The observations do not depend on cluster membership; hence, the traditional one-level regression analysis will be preferred. On the other hand, ICC = 1 indicates a perfect interdependence of residuals: The observations only vary between clusters [12]. The random effect estimates the amount of variation in the outcome variable being contributed to by between-household and between-cluster groupings. These variations were, then, used to compute the intraclass correlations for each survey year (ICC). The ICC is an estimate of the proportion of variation in the outcome (CK of HIV and AIDS) contributed by the variability between individuals nested in households at level one grouping and households nested in clusters at level two grouping.

## 3. Results

### 3.1. Distribution of Categories within Variables and CK of HIV and AIDS

Table 1 shows the percentages of Ghanaians with CK of HIV and AIDS from 1998 to 2014 and overall. Generally, it is observed that CK of HIV and AIDS was lower in 1998 than in the rest of the survey years. The Northern region recorded the lowest percentage of CK of HIV and AIDS for each survey year. In 1998, among all the 10 administrative regions in Ghana, Brong Ahafo recorded the highest percentage of CK of HIV and AIDS (17.51%) followed by the Greater Accra region which had 16.62% of its population having CK of HIV and AIDS. However, in 2003, Greater Accra topped with a percentage of 43.99% of CK of HIV and AIDS whereas Brong Ahafo followed with 41.71%. Surprisingly, in 2008, Brong Ahafo was the only region which experienced a drop in the percentage of CK of HIV and AIDS. The remaining nine regions experienced an increase in CK of HIV and AIDS. Additionally, in 2014, all the regions experienced a drop in CK of HIV and AIDS with the Upper East experiencing a heavy drop: from 40.92% to 28.10%. For the pooled dataset, Greater Accra recorded the highest percentage (44.18%) of CK of HIV and AIDS followed by the Brong Ahafo region (35.52%). The northern region recorded the lowest percentage of CK of HIV and AIDS (17.87%).

Individuals who live in urban areas, for all the surveys, recorded a higher CK of HIV and AIDS than those living in the rural areas. Also, persons who have never married obtained the highest percentage of CK of HIV and AIDS in both 2003 and 2008: 42.40%, and 43.18% respectively. In 1998, those who are married but not living together attained the highest percentage of CK of HIV and AIDS, 15.72%; however, in 2014, those who had divorced recorded the highest percentage (39.66%).

### 3.2. Trend and Percentage of Ghanaians with CK of HIV/AIDS

**Figure 1:**
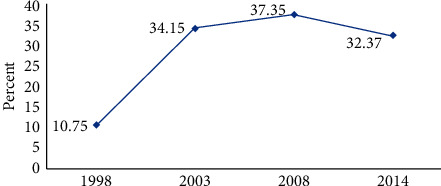
Trend of CK of HIV and AIDS among Ghanaians.

The trend and the prevalence of CK of HIV and AIDS are shown in Figure 1. Comprehensive knowledge of HIV and AIDS increased from 10.75 in 1998 to 34.15 in 2003. It also increased marginally from 34.15 in 2003 to 37.35 in 2008. The prevalence of CK of HIV and AIDS, however, experienced a decrease in 2014 from 37.35 in 2008 to 32.37. The percentage of CK of HIV and AIDS was also found from the pooled data (1998–2014) to be 32.50% with a 95% confidence interval of (31.51%, 33.55%).

### 3.3. Association between Time and CK of HIV and AIDS

Table 2 is the result from cross tabulation between survey years and CK of HIV and AIDS. A chi-square value of 1400 with a significant *p* value <0.001 found indicates a significant association between time (survey years) and CK of HIV and AIDS.

The association between time (survey year) and CK of HIV and AIDS was measured, and the result indicated that people in the survey year 2003 were 4.86 ([4.18, 5.66], *p* < 0.001) more likely to have CK of HIV and AIDS than those in 1998. Similarly, people in 2008 were 5.72 ([4.94, 6.61], *p* < 0.001) more likely to have CK of HIV and AIDS than those in 1998. Again, respondents in the survey year 2014 were found to be 4.55 ([3.90, 5.31], *p* < 0.001) more likely to have CK of HIV and AIDS than those in 1998. These associations were all found to be statistically significant with a *p* value<0.001.

### 3.4. Association between CK of HIV and AIDS and Independent Variables

The results of the Pearson chi-squared test of independence between CK of HIV and AIDS and the independent variables showed that a number of the variables were significantly associated with CK of HIV and AIDS across all survey years. In 1998, eleven of the variables showed statistical associations with CK of HIV and AIDS (*p* < 0.05), whereas in 2003, all the independent variables considered showed significant associations with the outcome variable (*p* < 0.05). However, in both 2008 and 2014 survey years, occupation was found not to be associated with CK of HIV and AIDS. Significant associations (*p* < 0.05) were also found between all predictors and CK when the test was performed on the pooled dataset, Table 3. Eleven variables, literacy, region, contraceptive method, place of residence, marital status, religion, education, newspaper, radio, television, and age of the household head, were found to be significantly associated with CK of HIV and AIDS across all the four survey years (*p* < 0.005).

### 3.5. Comparison of the Fixed Effect-Only Model to the Multilevel Model

A likelihood ratio test was performed to compare the multilevel logistic model to a standard binary logistic model. It tests the null hypothesis that the smaller model (fixed effect-only model) is better than the logistic regression model with the random effects (multilevel model). This test was run for all the survey years, and the results indicated that using a multilevel logistic model provides a better fit than using a standard logistic regression since a *p* value <0.001 was obtained for all the four models. The calculated *χ*^2^ for 1998 was 24.88, 2003 was 132.67, 2008 was 107.66, and 2014 was 167.05, all with a *p* value <0.001.

### 3.6. Multivariable Analysis of CK of HIV and AIDS

A multilevel (three-level) logistic regression model was used to analyze the effect of factors on CK on HIV and AIDS, and the results are presented in Table 4. The table presents the adjusted odds ratios and their 95% confidence intervals, as well as their *p* values. It also presents the variance of the random effects model with its confidence interval. The results show that the Upper East recorded the highest odds from 1998–2008 among all the 10 regions after adjusting for other covariates: 2.65 ([1.71, 4.10], *p* < 0.001) and 2.31 ([1.58, 3.40], *p* < 0.001) in 2003 and 2008, respectively, compared to the Western Region. However, in 2014, only two of these associations were significant: 1.55 ([0.17, 2.07], *p* < 0.01) for the Greater Accra region and 0.65 ([0.46, 0.93], *p* < 0.05) for the Upper West Region. With regards to the ethnicity, all the various ethnic groups were less likely to have CK of HIV and AIDS than Akans in 2008 and 2014 after adjusting for covariates, though only Gruma and Hausa were highly statistically significant.

With religion, the chance of having CK of HIV and AIDS was found to be higher among individuals who were affiliated to a religious group than those who were not after holding all other covariates constant. Though higher, statistical significance was observed for only the years 2008 and 2014.

After adjusting for other factors, the odds of having CK of HIV and AIDS were found to be 2 to 3 times more likely for 2003, 2008, and 2014 survey years between respondents whose highest level of education was either secondary or tertiary as against CK of HIV and AIDS. As expected, respondents who could read easily were more likely to have CK of HIV and AIDS compared to those who could not read easily after holding all other covariates constant with all survey years except 2008 being statistically significant. Respondents who used contraceptives, for instance in 1998, 2003, 2008, and 2014, were 75%, 30%, 4%, and 1% more likely to have CK of HIV and AIDS compared to their counterparts who were not using. These were only statistically significant for survey years 1998 and 2003. Also, respondents from rural areas were less likely except for 1998 to have CK of HIV and AIDS than those who were in the urban centers after adjusting for other factors. Media exposure has also been found to be significantly associated with CK of HIV and AIDS.

From Table 4, it can be seen that 1998 recorded the highest ICC for both cluster and household levels. About 19% of the chances of having CK of HIV and AIDS was explained by between-household differences and, conversely, 81% was explained by within-household differences. Also, only 7% of the total variation was accounted for by the cluster differences, whereas 93% was accounted for by within-cluster differences. In 2014, the ICC at the household level was found to be only 9%. This indicated that only 9% of the chances of having CK of HIV and AIDS was explained by between-household differences. On the other hand, 91% was explained by within-household differences. Similarly, the chance of having CK of HIV and AIDS that were explained by the cluster differences was merely 6% whereas 94% was contributed by the within-cluster differences.

## 4. Discussion

Trends in CK of HIV and AIDS experienced an increase from 10.75% in 1998 to 34.15% in 2003. Respondents in 2003 were found to be 4.86 more likely to have CK of HIV and AIDS than those in 1998. In 2008, CK of HIV and AIDS increased slightly with the odds of 5.72 compared to 1998. However, in 2014, CK of HIV and AIDS decreased from 37.35% in 2008 to 32.37% in 2014. The odds were found to be 4.55 times among respondents in 2014 than those in 1998. The reason for the decline could be the heavy reliance on donor funds for HIV and AIDS campaign programs which is unsustainable. The total expenditure on disease-related activities for 2014 was about $68,843,316. Sixty-six percent of this amount was contributed by multilateral organizations, 27% from the private sector, and the remaining 7% from the government [14].

The findings from this study show that there was a significant variation in the prevalence of comprehensive knowledge among Ghanaians over the years, that is, from 1998 to 2014. The year 1998 recorded the lowest prevalence of comprehensive knowledge 10.75% with the highest being 37.35% for 2008. The overall prevalence for the combined dataset was 32.50%.

The findings further reveal that the prevalence of CK of HIV and AIDS was found to be low among Ghanaian women and men, especially in 1998, with 10.81% and 11.95%, respectively. This was expressively below the percentage obtained from the extracted pooled data of 29.24% and 37.70% for women and men, respectively. There was a National response target on CK of HIV and AIDS aimed at achieving a prevalence of 80% among both men and women by 2015 [9]. This target could not have been achieved since this study found less than half of the population (32.37%) of Ghanaians having CK of HIV and AIDS as at 2014. Also, CK of HIV and AIDS was found to be significantly lower in women than in men. Analysis on the four survey years (1998–2014) revealed a prevalence of less than 40% for each survey year. Meanwhile, a similar study by Teshome et al. [15] found a prevalence of 48.90% among women in Burundi, 2010. The same study also found 46.30% of Kenyan women with CK of HIV and AIDS (2009). This finding is not surprising since women account for about two-thirds of the world's illiterate adults [16].

Comprehensive Knowledge of HIV and AIDS has also been found to be higher among people in urban areas than in rural areas (40.40% vs. 25.24%) in the extracted pooled data. The odds were 0.45 and 0.72 less in persons living in rural areas compared to those living in urban areas in the bivariate and multivariable models, respectively. This rural disadvantage also confirms an observation in [17]. An analogous study was conducted in Burkina Faso using the Burkina Faso DHS (2010). The disparity in CK of HIV and AIDS between rural and urban areas was found to be about 25.59%. This could be attributed to the low patronage of education in the rural areas than the urban areas. Studies have also revealed that one's education has a great role in determining the person's social status, access to information, and income. The study by Rahman [18] reveals that educated people had a higher chance of having CK of HIV and AIDS than those who had no education. The odds of having CK of HIV and AIDS was high for respondents with higher educational levels. A similar result was found by Ochako et al. [19], where the odds of having CK of HIV and AIDS was found to be as high as 17.52 among Kenyan women with secondary or higher education than in those with no education. The role of education in the lives of people may not only be providing people with information needed to guard themselves from getting infected but also motivating people to be interested in their own health and to take better care of their health for a successful future [20]. Access to formal education and sustained HIV and AIDS training and awareness was also recommended by Agyemang et al. in order to diffuse false prejudices and beliefs about HIV and AIDS [21].

Television, newspaper/magazine, and radio are effective media tools to reach the general population in order to communicate relevant information through music, news items, dramas, and advertisements. Significant associations have been found between media exposure and CK of HIV and AIDS in the pooled GDHS data. This study found out that persons watching television, at least, once a week had 2.11 and 1.36 times the odds of having CK of HIV and AIDS, in the bivariate and multivariable models, respectively, than those who did not watch television, at least, once a week. Similarly, respondents that read, at least, a newspaper once a week had higher odds of having CK than those who did not read a newspaper for, at least, a week. This is similar to a study by Sheikh [13], who found out that Bangladesh women exposed to the media had higher odds of being knowledgeable in HIV and AIDS than those who were not exposed to the media. Also, people in urban areas might be more exposed to the media and campaigns related to HIV and AIDS. According to a study by Veinot and Harris [22], differences in social networks could also be the reason for the urban-rural gap in HIV and AIDS knowledge. The electronic media is a prime source of information about HIV and AIDS [21].

The results show that respondents found to be working were 0.96 less likely to have CK of HIV and AIDS than those who were not working. Jha et al. [23], however, found that Indian women living in slum areas and in the working category were 1.29 times more likely to be knowledgeable in HIV and AIDS than those not working. For our study, the working group comprised of professionals, technicians, managers, clericals, sales persons, and skilled and unskilled manual. It is possible that the nonworking will mostly consist of young people who would have probably been schooling where they might be exposed to some pragmatic ways to avoid sexually transmitted diseases including HIV and AIDS.

## 5. Conclusions/Recommendations

This study sought to assess the trends and determinants of comprehensive knowledge (CK) of HIV and AIDS among Ghanaian adults using the Ghana Demographic Health Surveys (GDHS) from 1998 to 2014. The findings from this study highlight that the percentage of Ghanaians with CK of HIV and AIDS has decreased in 2014. The study also observed high disparities of CK of HIV and AIDS among the 10 administrative regions in the country. Comprehensive knowledge of HIV and AIDS was lower in the Northern region and relatively high in the Greater Accra region. Females were also found to have lower CK of HIV and AIDS than males. Although people who are employed are expected to have higher CK of HIV and AIDS than their counterparts who are not employed, the findings of this study indicate that individuals who are working have a lower CK of HIV and AIDS than those who are not. However, the difference in CK of HIV and AIDS between the two categories was marginal. Comprehensive knowledge of HIV and AIDS was found to be highest in 2008 among the four survey years (1998, 2003, 2008, and 2014). Moreover, CK of HIV and AIDS was significantly associated with media exposure, contraceptive use, gender, type of place of residence, and literacy and education.

Hence, education on prevention and transmission methods needs to be intensified in rural areas, as well as areas with no or little formal education. Educational campaigns on HIV and AIDS should also target regions with low HIV and AIDS knowledge such as the Northern and the Upper West regions to reverse some of the knowledge gaps and correct the local misconceptions. Mass media advertisement and sensitizations on prevention and transmission modes should also be encouraged.

## Figures and Tables

**Table 1:** Percentage distribution of categories within variables with comprehensive knowledge (CK) of HIV and AIDS.

Variables	1998	2003	2008	2014	All years
Region					
Western	10.93	34.36	34.70	34.17	29.90
Central	7.87	28.74	44.04	41.26	32.87
Greater Accra	16.62	43.99	52.86	49.92	44.18
Volta	7.92	32.55	39.13	25.97	27.26
Eastern	10.40	34.83	35.99	32.23	29.52
Ashanti	8.88	39.34	39.64	32.14	33.14
Brong Ahafo	17.51	41.71	39.54	34.24	35.52
Northern	3.04	18.63	21.16	17.92	17.87
Upper East	14.91	40.46	40.92	28.10	32.99
Upper West	3.18	20.39	27.39	18.66	19.04

Ethnicity					
Akan	11.70	38.99	42.53	38.94	35.26
Ga/Dangme	12.93	32.89	41.16	42.47	34.63
Ewe	10.45	33.23	38.86	35.05	30.88
Guan	8.00	47.21	37.81	31.89	36.05
Mole-Dagbani	10.46	28.85	33.90	27.74	28.34
Grusi	7.47	43.25	40.38	30.63	32.54
Gruma	10.51	17.09	20.89	15.02	16.05
Hausa	14.42	49.64	38.00	16.86	29.58
Others	6.20	30.63	40.11	34.12	28.10

Religion					
No religion/other	6.78	23.83	26.97	19.50	19.48
Catholic	12.43	37.80	40.80	35.29	33.08
Anglican	24.64	55.01	53.56	46.25	47.61
Methodist	14.55	38.18	41.24	41.56	35.44
Presbyterian	13.44	37.93	46.75	41.38	35.66
Traditionalist	8.21	19.68	23.20	11.38	15.97
Other Christian	10.54	38.19	41.53	36.95	34.85
Muslim	8.96	30.79	34.07	30.45	29.00

Education					
No education	5.75	19.70	20.51	15.25	15.88
Primary	6.61	22.37	27.77	21.22	20.84
Secondary	13.64	43.85	44.76	39.61	38.01
Higher	30.71	70.86	71.13	67.89	66.14

Occupation					
Not working	10.57	43.69	40.34	34.50	34.35
Working	11.24	33.47	39.10	35.34	32.04

Contraceptive					
Not using	9.34	33.10	37.52	34.11	30.59
Using	18.24	43.32	45.65	38.54	38.87

Place of residence					
Urban	13.81	44.87	48.11	40.88	40.4
Rural	9.52	27.47	31.28	28.47	25.24

Marital status					
Never married	10.13	42.40	43.18	38.73	37.22
Married	10.55	31.68	37.42	34.64	30.45
Living together	13.62	36.40	37.81	28.33	28.99
Widowed	6.13	25.78	30.98	30.05	25.80
Divorced	12.84	27.97	33.50	39.66	29.70
Not together	15.72	37.10	35.33	29.76	29.90
Variables	1998	2003	2008	2014	All years

Gender					
Male	11.95	39.82	42.44	39.40	37.7
Female	10.81	32.01	36.45	35.17	29.24

Reads newspaper					
No	8.04	29.94	34.80	32.44	28.83
Yes	20.26	58.46	56.55	55.80	49.13
Listens to radio					
No	6.13	20.93	28.47	29.56	23.2
Yes	13.81	38.96	41.61	38.73	36.08

Watches Tv					
No	7.08	27.10	28.08	28.88	24.67
Yes	14.76	45.15	47.69	40.13	39.36

Literacy					
Reads easily	14.61	50.64	52.59	47.26	43.09
Reads with difficulty	7.91	29.92	36.59	30.49	31.26
Cannot read	5.81	22.40	25.03	19.58	19.68

Age of the respondent					
15–19	9.26	37.67	37.42	30.20	30.86
20–24	12.21	41.39	44.15	37.15	35.64
25–29	12.11	36.46	43.54	41.20	35.62
30–34	11.04	32.36	41.02	38.06	32.89
35–39	12.80	30.44	35.57	36.16	30.95
40–44	8.45	34.38	35.09	30.89	29.37
45–49	11.04	30.37	32.77	30.08	27.98
50–54	10.16	39.68	41.76	36.73	36.76
55–59	14.26	39.28	42.34	32.16	35.23

Age of the HH head					
<20	6.64	25.65	35.01	24.76	23.15
20–29	13.52	38.58	43.82	39.95	36.07
30–39	11.94	33.58	39.12	37.11	32.79
40–49	8.84	34.61	36.18	33.17	30.63
50–59	13.29	40.74	42.45	33.10	34.37
60–69	8.30	32.57	37.82	33.88	30.38
70–79	8.77	34.55	35.38	29.84	29.52
Above 80	9.02	32.79	31.44	32.94	28.14

**Table 2:** Cross tabulation of survey years and CK of HIV and AIDS.

Year	CK of HIV/AIDS		Total	*χ* ^2^	*p* value
	Yes	No			
1998	657	5.455	6.112	1.400	<0.001
2003	3.598	6.938	10.536		
2008	3.482	5.842	9.324		
2014	4.344	9.076	13.420		
Total	12.081	27.311	39.392		

**Table 3:** CK of HIV and AIDS among Ghanaians (pooled dataset).

Variable	Bivariate			Multivariable		
	OR	95% CI		OR	95% CI	
		*L*	*U*		*L*	*U*
Region						
Western	1.00			1.00		
Central	1.96^*∗∗∗*^	1.60	2.40	1.25^*∗*^	1.03	1.52
Greater Accra	0.94	0.76	1.16	1.34^*∗∗*^	1.11	1.63
Volta	1.01	0.82	1.25	1.16	0.92	1.46
Eastern	1.21^*∗*^	1.02	1.44	1.02	0.83	1.24
Ashanti	1.35^*∗∗*^	1.10	1.67	1.11	0.94	1.31
Brong Ahafo	0.49^*∗∗∗*^	0.37	0.63	1.53^*∗∗∗*^	1.28	1.821
Northern	1.10	0.88	1.38	0.70^*∗∗*^	0.55	0.89
Upper East	0.58^*∗∗∗*^	0.44	0.75	1.68^*∗∗∗*^	1.33	2.13
Upper West	1.18	0.96	1.46	0.76^*∗*^	0.60	0.97

Ethnicity						
Akan						
Ga/Dangme	0.90	0.79	1.03	0.84^*∗*^	0.74	0.96
Ewe	0.80^*∗∗∗*^	0.72	0.90	0.86^*∗*^	0.76	0.97
Guan	0.99	0.79	1.25	1.31^*∗*^	1.06	1.62
Mole-Dagbani	0.68^*∗∗∗*^	0.60	0.77	1.24^*∗∗*^	1.06	1.45
Grusi	0.68^*∗∗∗*^	0.55	0.84	1.10	0.89	1.35
Gruma	0.32^*∗∗∗*^	0.25	0.41	0.77^*∗*^	0.61	0.97
Hausa	0.66^*∗*^	0.47	0.92	0.82	0.59	1.16
Others	0.83	0.61	1.14	0.96	0.79	1.18

Religion						
No/other				1.00		
Catholic	2.13^*∗∗∗*^	1.83	2.48	1.39^*∗∗∗*^	1.20	1.62
Anglican	3.69^*∗∗∗*^	2.77	4.92	1.92^*∗∗∗*^	1.44	2.57
Methodist	2.36^*∗∗∗*^	2.01	2.78	1.29^*∗∗*^	1.10	1.53
Presbyterian	2.47^*∗∗∗*^	2.08	2.94	1.40^*∗∗∗*^	1.18	1.65
Traditionalist	0.84	0.67	1.05	1.03	0.84	1.29
Other Christian	2.26^*∗∗∗*^	1.96	2.60	1.46^*∗∗∗*^	1.27	1.68
Moslem	1.66^*∗∗∗*^	1.40	1.98	1.42^*∗∗∗*^	1.19	1.69

Education						
No education				1.00		
Primary	1.50^*∗∗∗*^	1.35	1.67	1.21^*∗∗∗*^	1.09	1.34
Secondary	3.61^*∗∗∗*^	3.27	4.00	1.84^*∗∗∗*^	1.65	2.06
Higher	12.50^*∗∗∗*^	10.86	14.40	4.49^*∗∗∗*^	3.85	5.24

Occupation						
Not working						
Working	0.87^*∗∗∗*^	0.82	0.93	0.96	0.90	1.04

Contraceptive						
Not using						
Using	1.51^*∗∗∗*^	1.42	1.61	1.182^*∗∗∗*^	1.12	1.26

Place of residence						
Urban						
Rural	0.45^*∗∗∗*^	0.41	0.49	0.72^*∗∗∗*^	0.65	0.79

Marital status						
Never married						
Married	0.72^*∗∗∗*^	0.68	0.77	0.93	0.85	1.02
Living together	0.70^*∗∗∗*^	0.64	0.77	0.86^*∗∗*^	0.77	0.96
Widowed	0.52^*∗∗∗*^	0.43	0.63	0.85	0.68	1.07
Divorced	0.69^*∗∗∗*^	0.59	0.79	0.90	0.76	1.07
Not together	0.70^*∗∗∗*^	0.60	0.81	0.82^*∗*^	0.69	0.96

Gender						
Male						
Female	0.64^*∗∗∗*^	0.61	0.69	0.81^*∗∗∗*^	0.75	0.87

Reads newspaper						
No						
Yes	2.62^*∗∗∗*^	2.44	2.82	1.12^*∗∗*^	1.04	1.21

Listens to radio						
No						
Yes	1.96^*∗∗∗*^	1.82	2.11	1.31^*∗∗∗*^	1.22	1.41

Watches Tv						
No						
Yes	2.11^*∗∗∗*^	1.97	2.26	1.36^*∗∗∗*^	1.06	1.21

Literacy						
Reads easily						
Reads with difficult	0.55^*∗∗∗*^	0.50	0.60	0.76^*∗∗∗*^	0.69	0.83
Cannot read	0.28^*∗∗∗*^	0.26	0.31	0.62^*∗∗∗*^	0.57	0.68

Age of the respondent						
15–19						
20–24	1.27^*∗∗∗*^	1.17	1.37	1.25^*∗∗∗*^	1.15	1.38
25–29	1.28^*∗∗∗*^	1.17	1.39	1.37^*∗∗∗*^	1.22	1.53
30–34	1.08	0.99	1.19	1.32^*∗∗∗*^	1.15	1.51
35–39	0.93	0.85	1.02	1.23^*∗∗*^	1.08	1.41
40–44	0.92	0.83	1.02	1.27^*∗∗*^	1.09	1.49
45–49	0.85^*∗∗*^	0.77	0.94	1.21^*∗*^	1.05	1.4
50–54	1.36^*∗∗∗*^	1.17	1.59	1.25^*∗*^	1.02	1.52
55–59	1.16	0.96	1.41	1.01	0.78	1.29

Age of the HH head						
<20						
20–29	1.92^*∗∗∗*^	1.44	2.54	1.41^*∗*^	1.06	1.88
30–39	1.57^*∗∗*^	1.18	2.09	1.37^*∗*^	1.026	1.83
40–49	1.40^*∗*^	1.06	1.85	1.30	0.98	1.72
50–59	1.69^*∗∗∗*^	1.28	2.24	1.45^*∗*^	1.09	1.93
60–69	1.42^*∗*^	1.06	1.91	1.29	0.96	1.72
70–79	1.35	0.99	1.84	1.29	0.95	1.74
Above 80	1.29	0.89	1.86	1.38	0.96	1.97

*Note*. *p* < 0.05=^*∗*^, *p* < 0.01=^*∗∗*^, *p* < 0.001=^*∗∗∗*^, OR = odds ratio, *p* = probability value, Cl = confidence interval, *U* = upper limit, *L* = lower limit.

**Table 4:** Adjusted odds ratio of comprehensive knowledge of HIV and AIDS among Ghanaians (1998–2014).

Variable	1998		2003		2008		2014	
	AOR	95% CI		AOR	95% CI		AOR	95% CI		AOR	95% CI	
Region		*L*	*U*		*L*	*U*		*L*	*U*		*L*	*U*
Western	1.00			1.00			1.00			1.00		
Central	0.65	0.38	1.10	0.93	0.69	1.25	1.82^*∗∗∗*^	1.40	2.35	1.34	0.99	1.82
Greater Accra	1.03	0.64	1.67	0.96	0.70	1.32	1.77^*∗∗∗*^	1.36	2.31	1.55^*∗∗*^	0.17	2.07
Volta	0.65	0.34	1.23	1.09	0.76	1.55	1.91^*∗∗*^	1.27	2.88	0.82	0.61	1.10
Eastern	0.76	0.48	1.22	1.07	0.78	1.48	1.24	0.92	1.66	0.94	0.71	1.24
Ashanti	0.69	0.43	1.10	1.13	0.86	1.49	1.30^*∗*^	1.04	1.64	0.87	0.68	1.12
Brong Ahafo	1.95^*∗∗*^	1.22	3.12	1.64^*∗∗*^	1.19	2.25	1.51^*∗∗*^	1.12	2.04	1.31	0.99	1.72
Northern	0.27^*∗∗*^	0.12	0.62	0.60^*∗*^	0.38	0.96	0.77	0.54	1.10	0.77	0.55	1.06
Upper East	2.01	0.99	4.09	2.65^*∗∗∗*^	1.71	4.10	2.31^*∗∗∗*^	1.58	3.40	1.29	0.94	1.76
Upper West	0.47	0.18	1.21	0.72	0.49	1.06	1.10	0.77	1.57	0.65^*∗*^	0.46	0.93

Ethnicity												
Akan	1.00			1.00			1.00			1.00		
Ga/Dangme	0.93	0.60	1.43	0.80	0.63	1.00	0.88	0.69	1.14	0.99	0.82	1.20
Ewe	1.05	0.67	1.37	0.82	0.65	1.03	0.81^*∗*^	0.66	0.99	0.92	0.78	1.09
Guan	1.03	0.37	2.86	1.72^*∗∗*^	1.25	2.37	1.07	0.69	1.65	0.86	0.62	1.20
Mole-Dagbani	1.50	0.81	2.75	1.12	0.84	1.49	1.06	0.81	1.38	0.92	0.76	1.12
Grusi	0.66	0.28	1.60	1.34	0.90	2.00	1.28	0.85	1.94	0.93	0.71	1.22
Gruma	1.52	0.71	3.27	0.85	0.54	1.34	0.74	0.42	1.30	0.59^*∗∗∗*^	0.44	0.79
Hausa	1.52	0.69	3.33	1.49	0.85	2.62	1.23	0.59	2.54	0.44^*∗∗∗*^	0.29	0.67
Others	0.73	0.38	1.43	1.09	0.77	1.53	1.05	0.77	1.44	0.74	0.53	1.03

Religion												
No/other	1.00			1.00			1.00			1.00		
Catholic	1.34	0.83	2.17	1.16	0.89	1.50	1.24	0.94	1.65	1.72^*∗∗∗*^	1.30	2.29
Anglican	2.37	0.89	0.63	1.78^*∗∗*^	1.02	3.10	1.63	0.97	2.76	1.86^*∗*^	1.11	3.12
Methodist	1.38	0.81	2.36	1.15	0.85	1.57	1.09	0.79	1.51	1.50^*∗*^	1.10	2.04
Presbyterian	1.18	0.69	2.01	1.03	0.76	1.38	1.38	1.00	1.90	1.84^*∗∗∗*^	1.35	2.52
Traditionalist	1.17	0.66	2.06	0.88	0.61	1.26	1.18	0.82	1.70	1.26	0.84	1.91
Other Christian	1.06	0.67	1.66	1.30^*∗*^	1.01	1.66	1.24	0.95	1.61	1.64^*∗∗∗*^	1.26	2.15
Muslim	1.17	0.66	2.08	1.03	0.76	1.39	1.21	0.88	1.66	1.93^*∗∗∗*^	1.43	2.59

Occupation		*L*	*U*		*L*	*U*		*L*	*U*		*L*	*U*
Not working	1.00			1.00			1.00			1.00		
Working	1.23	0.92	1.65	0.79^*∗∗*^	0.68	0.92	1.15	0.98	1.35	0.95	0.85	1.07

Contraceptive												
Not using	1.00			1.00			1.00			1.00		
Using	1.75^*∗∗∗*^	1.40	2.19	1.30^*∗∗∗*^	1.16	1.47	1.04	0.92	1.18	1.01	0.91	1.11

Place of residence												
Urban	1.00			1.00			1.00			1.00		
Rural	1.15	0.87	1.51	0.61^*∗∗∗*^	0.52	0.72	0.76^*∗∗∗*^	0.65	0.88	0.87^*∗*^	0.77	0.99

Marital status												
Never married	1.00			1.00			1.00			1.00		
Married	1.41	1.00	2.01	0.99	0.84	1.18	1.07	0.89	1.29	1.00	0.87	1.16
Living together	1.62^*∗*^	1.07	2.43	1.10	0.85	1.43	1.14	0.92	1.42	0.78^*∗∗*^	0.65	0.92
Widowed	0.98	0.37	2.65	0.96	0.59	1.57	0.99	0.63	1.55	0.89	0.62	1.25
Divorced	2.29^*∗∗*^	1.32	3.96	0.82	0.58	1.16	1.09	0.76	1.55	1.01	0.75	1.35
Not together	2.33^*∗∗*^	1.39	3.92	1.03	0.78	1.36	0.93	0.66	1.29	0.74^*∗*^	0.57	0.95

Gender												
Male	1.00			1.00			1.00			1.00		
Female	1.37^*∗*^	1.04	1.80	0.92	0.81	1.05	0.86^*∗*^	0.75	0.98	0.88	0.78	1.00

Reads newspaper												
No	1.00			1.00			1.00			1.00		
Yes	2.00^*∗∗∗*^	1.55	2.58	1.41^*∗∗∗*^	1.22	1.63	1.09	0.95	1.26	1.02^*∗∗*^	1.09	1.44

Listens to radio												
No	1.00			1.00			1.00			1.00		
Yes	1.55^*∗∗∗*^	1.22	1.96	1.37^*∗∗∗*^	1.67	1.61	1.21^*∗*^	1.03	1.41	1.06	0.96	1.18

Watches Tv												
No	1.00			1.00			1.00			1.00		
Yes	1.42^*∗∗*^	1.13	1.78	1.10	0.96	1.25	1.34^*∗∗∗*^	1.19	1.52	1.09	0.98	1.22

Literacy		*L*	*U*		*L*	*U*		*L*	*U*		*L*	*U*
Reads easily	1.00			1.00			1.00			1.00		
Difficulty in reading	0.47	0.12	1.78	0.52^*∗∗∗*^	0.44	0.62	0.66^*∗∗∗*^	0.56	0.78	0.58^*∗∗∗*^	0.50	0.68
Cannot read	0.39	0.11	1.41	0.53^*∗∗∗*^	0.48	0.67	0.51^*∗∗∗*^	0.43	0.60	0.42^*∗∗∗*^	0.37	0.49

Age of the respondent												
15–19	1.00			1.00			1.00			1.00		
20–24	0.92	0.63	1.34	1.26^*∗*^	1.05	1.50	1.29^*∗∗*^	1.07	1.56	1.42^*∗∗∗*^	1.23	1.64
25–29	0.81	0.53	1.25	1.29	0.91	1.40	1.37^*∗∗*^	1.10	1.71	1.84^*∗∗∗*^	1.53	2.22
30–34	0.71	0.44	1.14	1.06	0.82	1.37	1.32^*∗*^	1.01	1.72	1.88^*∗∗∗*^	1.52	2.34
35–39	0.82	0.52	1.29	0.93	0.72	1.21	1.13	0.86	1.48	1.72^*∗∗∗*^	1.39	2.13
40–44	0.60	0.54	1.01	1.27	0.97	1.68	1.04	0.77	1.40	1.64^*∗∗∗*^	1.29	2.08
45–49	0.88	0.52	1.50	1.01	0.77	1.33	0.96	0.72	1.28	1.53^*∗∗*^	1.20	1.95
50–54	0.45	0.17	1.17	0.97	0.67	1.40	0.88	0.60	1.27	1.89^*∗∗∗*^	1.34	2.65
55–59	0.85	0.33	2.19	0.79	0.51	1.24	0.80	0.51	1.24	1.34^*∗*^	0.90	2.00

Age of the HH head												
<20	1.00			1.00			1.00			1.00		
20–29	2.26	0.86	5.95	1.15	0.81	2.91	0.89	0.57	1.39	1.37	0.77	2.43
30–39	2.04	0.79	5.31	1.49	0.78	2.82	0.86	0.56	1.32	1.22	0.69	2.14
40–49	1.48	0.57	3.83	1.35	0.72	2.54	0.87	0.57	1.33	1.21	0.69	2.10
50–59	2.18	0.85	5.60	1.75	0.92	3.33	1.03	0.68	1.57	1.24	0.71	2.18
60–69	1.35	0.50	3.62	1.31	0.68	2.51	0.84	0.54	1.30	1.26	0.71	2.23
70–79	1.99	0.71	5.55	1.39	0.72	2.68	0.78	0.49	1.25	1.11	0.60	2.05
Above 80	1.93	0.45	8.34	1.28	0.61	2.68	0.68	0.34	1.35	1.51	0.75	3.04

Education												
No education	1.00			1.00			1.00			1.00		
Primary	1.07	0.68	1.68	0.90	0.76	1.07	1.23	1.01	1.50	1.33^*∗∗*^	1.11	1.59
Secondary	0.76	0.20	2.79	1.47^*∗∗∗*^	1.22	7.79	1.70^*∗∗∗*^	1.38	2.09	1.70^*∗∗∗*^	1.41	2.05
Higher	1.55^*∗∗∗*^	0.41	5.88	2.80^*∗∗∗*^	2.05	3.84	3.67^*∗∗∗*^	2.69	5.00	3.56^*∗∗∗*^	2.81	4.50
Constant	0.02	0.00	0.09	0.27^*∗∗*^	0.13	0.57	0.23^*∗∗∗*^	0.13	0.41	0.21^*∗∗∗*^	0.08	0.29
ICC												
Clust no.	0.07	0.04	0.12	0.06	0.04	0.08	0.06	0.04	0.08	0.06	0. 05	0.08
Clust no. > HH no.	0.19	0.10	0.34	0.13	0.10	0.17	0.11	0.08	0.16	0.09	0.06	0.14

*Note*. *p* < 0.05=^*∗*^, *p* < 0.01=^*∗∗*^, *p* < 0.001=^*∗∗∗*^, OR = odds ratio, *p* = probability value, Cl = confidence interval, *U* = upper limit, *L* = lower limit.

## Data Availability

The datasets generated and/or analyzed during the current study are available in the Ghana demographic and health survey repository, http://dhsprogram.com/data/available-datasets.cfm.
